# *Trypanosoma cruzi* loop-mediated isothermal amplification (*Trypanosoma cruzi* Loopamp) kit for detection of congenital, acute and Chagas disease reactivation

**DOI:** 10.1371/journal.pntd.0008402

**Published:** 2020-08-14

**Authors:** Susana A. Besuschio, Albert Picado, Arturo Muñoz-Calderón, Diana P Wehrendt, Marisa Fernández, Alejandro Benatar, Zoraida Diaz-Bello, Cecilia Irurtia, Israel Cruz, Joseph M Ndung’u, María L Cafferata, Graciela Montenegro, Sergio Sosa Estani, Raúl H. Lucero, Belkisyole Alarcón de Noya, Silvia A Longhi, Alejandro G Schijman

**Affiliations:** 1 Laboratorio de Biología Molecular de la Enfermedad de Chagas, Instituto de Investigaciones en Ingeniería Genética y Biología Molecular “Dr Héctor Torres”, (INGEBI-CONICET), Buenos Aires, Argentina; 2 Foundation for Innovative New Diagnostics (FIND), Geneva, Switzerland; 3 Hospital de Enfermedades Infecciosas “Dr. Francisco J. Muñiz” Buenos Aires, Argentina; 4 Instituto Nacional de Parasitología, “Dr Mario Fatala Chabén”, ANLIS CG Malbrán, Buenos Aires, Argentina; 5 Instituto de Medicina Tropical, Universidad Central de Venezuela, Caracas, Venezuela 5; 6 Hospital Nacional “Profesor Alejandro Posadas”, Villa Sarmiento, Buenos Aires, Argentina; 7 National School of Public Health, Instituto de Salud Carlos III, Madrid, Spain; 8 Departamento en Salud de la Madre y el Niño, Instituto de Efectividad Clínica y Sanitaria – Centro de Investigación en Epidemiología y Salud Pública (IECS-CIESP), Buenos Aires, Argentina; 9 Área de Biología Molecular, Instituto de Medicina Regional, Universidad Nacional del Nordeste, Resistencia, Argentina; University of Georgia, UNITED STATES

## Abstract

A *Trypanosoma cruzi* Loopamp kit was recently developed as a ready-to-use diagnostic method requiring minimal laboratory facilities. We evaluated its diagnostic accuracy for detection of acute Chagas disease (CD) in different epidemiological and clinical scenarios. In this retrospective study, a convenience series of clinical samples (venous blood treated with EDTA or different stabilizer agents, heel-prick blood in filter paper or cerebrospinal fluid samples (CSF)) from 30 infants born to seropositive mothers (13 with congenital CD and 17 noninfected), four recipients of organs from CD donors, six orally–infected cases after consumption of contaminated guava juice and six CD patients coinfected with HIV at risk of CD reactivation (N = 46 patients, 46 blood samples and 1 CSF sample) were tested by *T*. *cruzi* Loopamp kit (*Tc* LAMP) and standardized quantitative real-time PCR (qPCR). *T*. *cruzi* Loopamp accuracy was estimated using the case definition in the different groups as a reference. Cohen’s kappa coefficient (κ) was applied to measure the agreement between *Tc* LAMP (index test) and qPCR (reference test). Sensitivity and specificity of *T*. *cruzi* Loopamp kit in blood samples from the pooled clinical groups was 93% (95% CI: 77–99) and 100% (95% CI: 80–100) respectively. The agreement between *Tc* LAMP and qPCR was almost perfect (κ = 0.92, 95% CI: 0.62–1.00). The *T*. *cruzi* Loopamp kit was sensitive and specific for detection of *T*. *cruzi* infection. It was carried out from DNA extracted from peripheral blood samples (via frozen EDTA blood, guanidine hydrochloride-EDTA blood, DNAgard blood and dried blood spots), as well as in CSF specimens infected with TcI or TcII/V/VI parasite populations. The *T*. *cruzi* Loopamp kit appears potentially useful for rapid detection of *T*. *cruzi* infection in congenital, acute and CD reactivation due to HIV infection.

## Author summary

The aim of this study was to evaluate a kit prototype (*T*. *cruzi* Loopamp or *Tc* LAMP) based on loop-mediated isothermal amplification (LAMP) for molecular diagnosis of acute Chagas disease in well-characterized individuals, collected in real life conditions, such as newborns or infants born to Chagas disease mothers aiming to detect congenital Chagas disease, recipients of organs from Chagas disease donors who acquired *T*. *cruzi* infection after transplantation, persons with oral Chagas disease acquired after consumption of a contaminated meal, and HIV/*T*. *cruzi* coinfected patients at risk of Chagas disease reactivation due to immunosuppression. Different types of clinical samples were tested; peripheral blood samples treated with ethylenediamine tetraacetic acid (EDTA) as anticoagulant or with guanidine hydrochloride or DNAgard stabilizer agents, as well as dried blood spots and cerebrospinal fluid samples. The performance of *Tc* LAMP was estimated using the case definition in the different groups as a reference and all test kit results were compared to those obtained using standardized duplex real-time PCR (qPCR) in the same clinical samples. Sensitivity and specificity of *Tc* LAMP in blood samples from the pooled clinical groups were 93% (95% CI: 77–99) and 100% (95% CI: 80–100) respectively. The agreement between *Tc* LAMP and qPCR was almost perfect (κ index of Cohen = 0.92, 95% CI: 0.62–1.00). *Tc* LAMP was sensitive and specific for detection of *T*. *cruzi* infection in the tested samples, encouraging prospective field studies to validate its use for rapid detection of *T*. *cruzi* infection in congenital, acute and CD reactivation due to HIV infection.

## Introduction

Chagas disease (CD), also known as American trypanosomiasis, is a neglected tropical disease caused by the protozoan parasite *Trypanosoma cruzi* that affects about 6 to 7 million people worldwide, mainly in endemic areas of 21 Latin American countries [1].

Transmission of *T*. *cruzi* occurs by the vectorial route, including oral transmission by consuming food or beverages contaminated with triatomine faeces, by congenital transmission, blood transfusion or solid organ transplantation from infected donors, and by laboratory accidents.

The disease evolves from an acute phase when the infection is acquired, which is frequently asymptomatic, to a chronic phase that may develop, in up to 30% of cases, cardiac disease, and in 10% of cases digestive mega-syndromes, neurological and/or mixed complications [2,3]. A proportion of chronically yet asymptomatic infected people, facing an immunocompromised condition due to HIV infection, organ transplantation, autoimmune disease, or oncologic treatments, may experience CD reactivation, evolving to severe clinical forms of the disease with high parasitemia [4].

Early diagnosis of infection is crucial in cases of any acute infections, because it allows prompt and timely trypanocidal treatment, reducing morbimortality of severe clinical presentations of CD and progression to symptomatic chronic infections [5–8]. However, traditional parasitological methods currently used for diagnosis in the abovementioned clinical settings have poor performance. In fact, microscopy observation is highly operator dependent and low sensitive, haemoculture and xenodiagnoses are cumbersome methods that may take several months before a result can be reported. On the other hand, serological assays for *T*. *cruzi* infection are not always applicable; for serodiagnosis of infants born to seropositive mothers it is necessary to wait until at least nine months of age [9], and in severely immunocompromised CD patients, false seronegative results may be obtained [4].

Molecular methods, particularly polymerase chain reaction (PCR), have been employed as an alternative to improving current diagnostic algorithms; however, most rural endemic areas do not have the infrastructure needed for PCR analysis [7,10–12]. In this context, the loop-mediated isothermal amplification method (LAMP) may offer the advantage of PCR but only requiring simple laboratory manipulations without the need for expensive equipment [13–15]. The feasibility to use a LAMP prototype kit for detection of acute *T*. *cruzi* infections has been reported [15].

The present study aimed to evaluate the performance of *T*. *cruzi* Loopamp in panels of clinical samples from acute CD patients belonging to clinical settings of high vulnerability, in which an accurate and easy to handle infection detection method could significantly improve patient diagnosis [10,11].

## Methods

### Ethics statement

Informed written consent was obtained from seropositive mothers, whose infants´ samples were included in the study after receiving permission from the institutional review board (IRB) of the Centro de Educación Médica e Investigaciones Clínicas “Norberto Quirno” (CEMIC), Buenos Aires, IRB 00001745- ORG 0001315, HHS, 10 July 2013). In the case of Chagas-HIV patients, the use of samples was authorized by the IRB of the Hospital A. Posadas and Hospital de Enfermedades Infecciosas “Dr. Francisco J. Muñiz” without written consent from patients (IRB notes NI4015/17 y NI012/18), in agreement with Argentine legislation in force at this time (Blood Donation Law N° 22990, Res. N°1409/15).

The use of archival samples from recipients of organs from infected donors was authorized by the IRB committees of Hospital Británico, Hospital Austral, and Hospital Alemán of Buenos Aires because permission to use samples for research purposes was included in the written informed consent form signed by patients when admitted for routine medical checkups.

The use of archival samples from cases of CD outbreak via oral transmission at Chichiriviche de la Costa, Venezuela was authorized by the Scientific Ethics Committee of the “IMT, Universidad Central de Venezuela”, as reported in Noya et al. [16]. After reading the terms of free and informed consent, each patient or legal representative signed the form and agreed to sample collection and physical and other para-clinical exams.

### Clinical samples

*Tc* LAMP (index test) was compared with standardized qPCR (comparator test) using panels of archival DNA samples that were extracted from clinical specimens of patients affected by congenital, acute *T*. *cruzi* infections or Chagas disease reactivation, belonging to the following groups:

#### Group I: Congenital CD (cCD)

This panel was formed of archival DNA from blood samples collected at the time of diagnosis from 30 babies (9 newborns and 21 infants) born to seropositive mothers in Argentina between 2013 and 2017. According to the gold standard diagnosis, i.e. parasitaemia detected by micromethod during the first months of life and/or positive serology during 10–12 months of life, 13 (43.3%) babies were confirmed as cCD and 17 (56.7%) were not infected by *T*. *cruzi*. All cCD cases detected by means of gold standard diagnosis were referred for treatment with Benznidazole following national guidelines [17]. In 26 cases, 1.5 mL of venous blood (VB) was collected by venepuncture in EDTA vacuum tubes and 1 mL VB was transferred to microtubes containing 250 μL of a commercial stabilizer (DNAgard, Biomatrica, San Diego, USA, proportion 1:4) for molecular analysis. The mixture was conserved at room temperature for no more than one year and afterwards it was stored at 4°C for a maximum period of two years, until DNA extraction, which was then stored at -20°C up until doing qPCR and *Tc* LAMP analyses. In the other four cCD newborns (Hospital A. Posadas, Buenos Aires), heel-prick dried blood spot (DBS) samples were collected onto Whatman 903 filter papers following the guidelines of the national program for screening diseases in newborns (Argentine Law 23.413/23.874).[18] Each DBS card [19] contained six spots with around 50 μL of heel-prick blood each. The cards were dried for at least 3 hours after blood collection and packed individually in zipped bags to be transported by courier at room temperature to the molecular biology laboratory. Upon reception, the zipped bags were stored at -20°C between 5 and 18 months until molecular analysis.

The micromethod was performed as described by Rissio et al. [20] and the serological analyses were carried out using two different commercial assays following the recommendations of the manufacturers, namely, Chagatest ELISA recombinante v.3.0 (Wiener Lab, Rosario, Argentina) and Indirect Hemagglutination (IHA), Chagatest HAI (Wiener Lab, Rosario, Argentina). Because at least two positive serological tests are necessary to consider a patient to be infected, as recommended by WHO and PAHO [21], in cases of inconclusive serological results, an indirect immunofluorescence (IFI, Inmunofluor Chagas, Biocientífica, S.A., Argentina) test was performed, following manufacturer instructions.

#### Group II: Orally-transmitted CD (oCD)

Blood samples from six patients residing in Chichiriviche de la Costa, Vargas State, Venezuela, who were diagnosed with orally-transmitted CD after consumption of *T*. *cruzi*-contaminated guava juice in March 2009, were evaluated by *Tc* LAMP and qPCR. The patients were admitted and followed up at the “Instituto de Medicina Tropical”, “Universidad Central de Venezuela”, Caracas, Venezuela[16]. Parasitological diagnosis was done either by microscopical search of trypomastigotes in peripheral blood or by parasite culture, and serological analysis was performed by in-house assays (ELISA and IHA) with a *T*. *cruzi* epimastigotes delipidized antigen for the detection of anti-human IgG and IgM. Clinical examination included electrocardiogram and echocardiography. The patients received supervised treatment with 7mg/kg/day of Benznidazole for 60 days. For molecular diagnosis, 5 mL of blood samples were collected in 5 mL of Guanidine hydrochloride solution, 6M EDTA 0.2 M, pH 8.00 (GE) and stored at 4°C.

#### Group III: Organ recipients from infected donors (Tx-RID)

The Tx-RID group was composed of four seronegative individuals who received organ transplants (kidney and/or liver) from *T*. *cruzi*-infected donors. Their infection was diagnosed by qPCR and/or Strout analyses, as described previously by Cura et al. [6]. All Tx-RID patients were treated with trypanocidal drugs, following the recommendations of the Argentinean Society of Infectiology [22]. For the molecular analysis of samples obtained between 2015–2017 in Buenos Aires, 5 mL of blood were collected in EDTA vacuum tubes and stored at -20°C during a maximum period of three years until processing for qPCR and *Tc* LAMP was done.

#### Group IV: Chagas disease and HIV coinfected patients (Chagas-HIV)

Seven clinical samples—EDTA-treated blood (EB, n = 2), guanidine hydrochloride-EDTA blood (GEB, n = 1), peripheral blood stored on Whatman 903 filter paper spots (DBS, n = 3) and cerebrospinal fluid samples (CSF, n = 1)—were obtained from six *T*. *cruzi*-HIV chronically coinfected patients with confirmed diagnosis for central nervous system (CNS) CD reactivation. The patients were admitted and clinically monitored at the Hospitals Francisco Muñiz and Alejandro Posadas, Buenos Aires, between 2014 and 2018. Diagnosis included microscopical analysis of the CSF specimens, use of the Strout method (parasitological blood test), and CNS imaging. In addition, in three patients admitted at Hospital Posadas, conventional satellite DNA and kDNA-based PCR tests were performed from DBS samples. The patients were treated with 5–7 mg/kg/day of Benznidazole. The CSF sample was collected in a microtube with O-ring caps and stored at -20°C during a maximum period of two years.

### Molecular detection of *T*. *cruzi* DNA

The *Tc* LAMP prototype was compared with standardized qPCR in panels of DNA samples extracted from clinical specimens of patients belonging to the clinical groups described above [10].

#### DNA extraction from clinical specimens

Nucleic acids were extracted from 200 μL aliquots of EDTA-blood or CSF samples, or from 300 μL of guanidine hydrochloride-EDTA blood or DNAgard blood using the High Pure PCR Template Preparation Kit (Roche, Germany). Before lysis, 5μL of 40 pg/μL linearized pZero plasmid was added as an internal amplification standard except for the oCD samples; each sample was then purified following manufacturer recommendations [23]. DNA extraction from DBS was carried out following the method reported by Walter and coworkers using the Roche kit [24]. For each patient card, three punches (3 mm diameter each) were taken and pooled into a sterile Eppendorf tube and 200 μL of lysis buffer were added. Following incubation with proteinase K for 3 hours at 55°C, DNA extraction was done according to manufacturer instructions and 50 μL of eluate was obtained. The DNA extracts from clinical samples were conserved at -20°C until their use for *Tc* LAMP and qPCR.

#### *Trypanosoma cruzi* prototype kit

*Tc* LAMP (index test) was the prototype kit developed by Eiken Chemical Co., Ltd. Japan, using as molecular target the repetitive satellite DNA sequence of *T*. *cruzi* as previously reported [15]. The reagents are in dried form on the inside of the cap of the reaction tube and are stable for one year at 30°C.

The LAMP reaction was standardized from 5 μl of DNA lysates from Group cCD patients due to sample volume restrictions in studies of newborns, while for the remaining groups in the study, the maximum volume recommended by the manufacturer (30 μl of DNA eluate) was used [15].

Each LAMP tube was flicked down to collect the solution at the bottom and placed upside down for two minutes to reconstitute the dried reagent, inverted five times to mix the contents followed by a spin down. The reaction tube contains calcein in a quenched state, bound to manganese ions. Once the LAMP reaction starts, pyrophosphate ions that are generated bind to the manganese ions, so calcein is released generating fluorescent light. Incubation of the reaction was carried out at 65°C for 40 minutes for isothermal amplification, followed by a step at 80°C for five minutes for enzyme inactivation using a Rotor Gene 6000 thermocycler (Corbett Life Science, Cambridgeshire, UK).

LAMP results are qualitative and are therefore expressed as positive (Pos) and negative (Neg), based on direct eye visualization [15]. One replicate was carried out per clinical sample. Sample panels were masked, and a series of six samples each were randomly chosen from all the panels to perform a LAMP round. Each round included a negative control (non-template control (NTC), distilled water) provided in the kit and a positive control (30 μL of 1fg/μL CL Brener stock (Tc VI) DNA). A LAMP operator read the results blinded to qPCR results and to the clinical data.

#### Quantitative real-time PCR (qPCR)

Duplex real-time qPCR using TaqMan probes targeted to *T*. *cruzi* satellite DNA, plus an internal amplification control (IAC), was carried out in an ABI7500 thermocycler (Applied Biosystems, Foster City, CA, USA) following standardized conditions previously reported [10,11].

All reactions included a strong positive control (SPC): 10 fg/ul of CL Brener DNA and a weak positive control (WPC): 1 fg/uL of CL Brener DNA. The qPCR results were expressed as positive if the cycle threshold (Ct) was lower than 40 and negative or non-detectable if no Ct was obtained. The qPCR experiments were done on duplicates per each DNA extract and the mean value of parasitic load from the duplicates was obtained. An indeterminate result for the qPCR was defined if IAC amplification gave an outlier Ct value after application of Tukey criteria, as reported by Duffy et al. [10]. Parasitic load was expressed in parasite equivalents/mL of sample (par. eq/mL). Laboratory operators of the qPCR assay were blinded to index test results and assayed a series of ten to twelve DNA samples per round. The research evaluator had no access to the *Tc* LAMP results nor to clinical information before the end of sample processing and reporting.

#### Quality control samples

In order to establish standard curves for qPCR, human seronegative blood samples (certified by a blood bank) were spiked with 0.5, 1, 10, 100, 1000, 10000 cultured cells of CL Brener (TcVI) for cCD, Tx-RID, Chagas-HIV groups because TcII/V/VI populations have been identified in most patients residing in Argentina [23, 25–28]. The TcVI strain harbors a similar copy dosage of the satellite DNA target sequence than TcII and TcV strains [23]. In contrast, seronegative blood spiked with 0.5, 1, 10, 100, 1000, 10000 Silvio X-10 (TcI) cultured cells was used for quantification of oCD group because TcI is the prevailing discrete typing unit (DTU) in Venezuela and harbors around one order of magnitude fewer number of copies than TcII, V and VI strains [29]. DNAgard seronegative-spiked blood was used to quantify cCD group samples, except those collected in DBS, which were not quantitated. Guanidine hydrochloride EDTA seronegative spiked blood was used for samples collected in guanidine hydrochloride EDTA buffer (oCD and some Chagas-HIV samples). The oCD and DBS samples were checked for DNA integrity. The oCD samples were tested using TaqMan RNase P Detection Reagents Kit (Thermo Fisher Scientific, Waltham, Massachusetts USA), following the guidelines of the manufacturer. The DNA quality of the DBS samples was checked by conventional amplification of β-actin gene, using primers ACT forward 5’-GGACCTGACTGACTACCTCATGGA-3 and ACT reverse 5’-GATCCACATCTGCTGGAAGGTGG-3’ to obtain a 550 bp fragment. Reaction was carried out in 25 μL of final volume containing 1X buffer, deoxynucleotide triphosphate (dNTP) 0.2 mM, 0.4 μM primers, MgCl2 2.5 mM, Taq Polymerase 1U and 5 μL of DNA eluate. Cycling was a first cycle at 94°C for 45 sec. followed by 35 cycles at 94°C for 20 sec, 62°C for 10 sec and 72°C for 20 sec, with a final step at 72°C for 45 sec.

### Statistical analysis

The *Tc* LAMP and qPCR data obtained from the blood samples (venous blood treated with EDTA or different stabilizer agents, heel-prick blood in filter paper) from the different groups of patients were pooled to estimate the accuracy of the *T*. *cruzi* Loopamp kit and the agreement between *Tc* LAMP and qPCR. The CSF samples were excluded from these analyses. The sensitivity and specificity of *Tc* LAMP were estimated using the case definition in the different groups as a reference (e.g. microscopy observation using the micromethod and serology for cCD; microscopy observation using the micromethod, culture and serology for oCD, and qPCR and/or Strout analysis for Tx-RID and Chagas-HIV patients). Cohen’s kappa coefficient (κ) was applied to measure the agreement between Tc LAMP and qPCR. The sensitivity and specificity estimates and their 95% exact binomial confidence intervals, as well as the Cohen kappa statistics, were calculated using the package epiR in R 3.5.0 [30].

## Results

### Comparison of *Tc* LAMP and qPCR in paired samples for detection of *T*. *cruzi* infection

**Fig 1:**
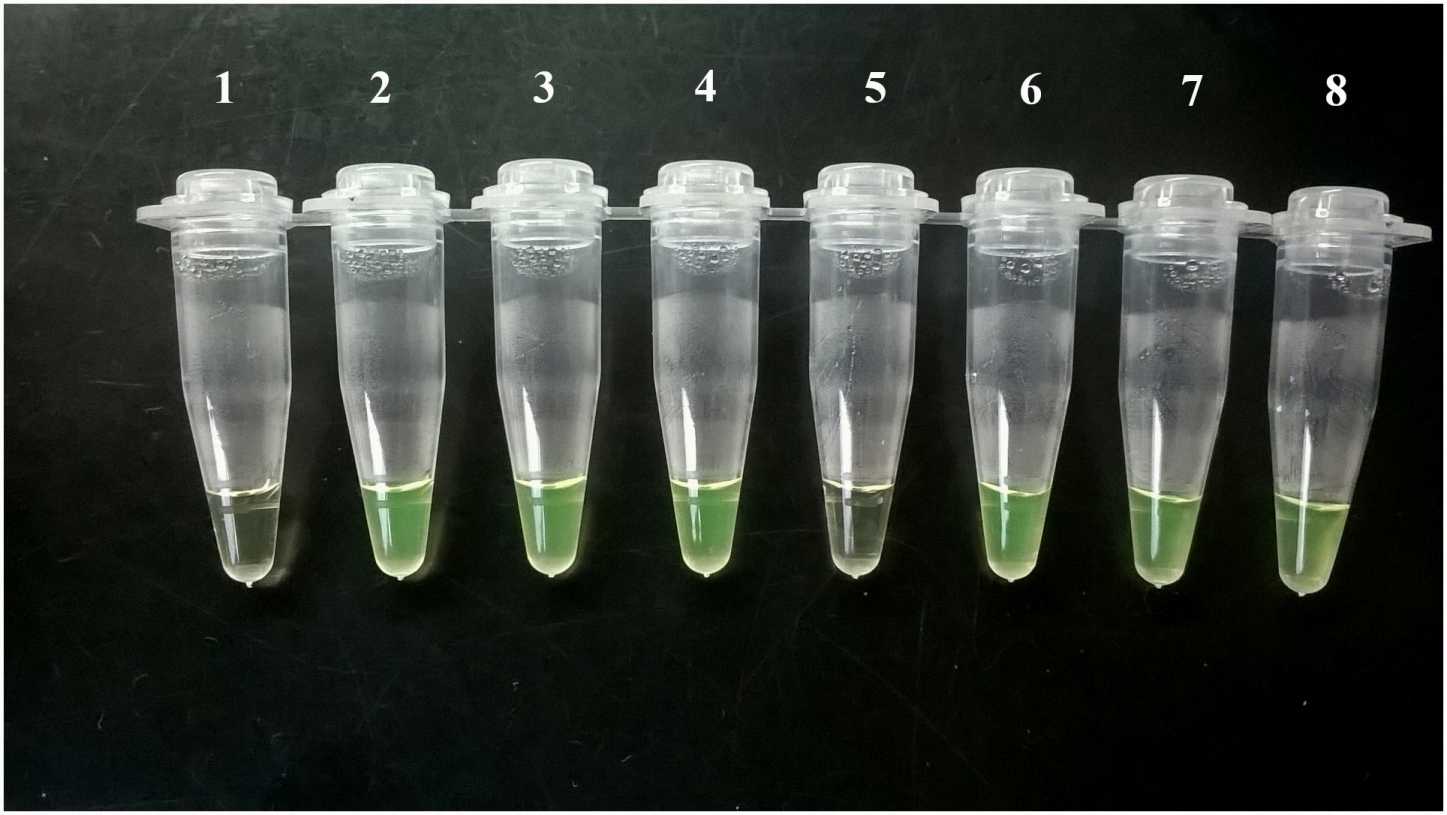
Visualization of Loopamp *Trypanosoma cruzi* results by the naked eye. Tube 1: NTC control: Tc LAMP reagents with distilled water and no template; tubes 2–4: cCD cases 1, 5 and 9 (Table 1); tubes 5–6: oCD cases 5 and 6 (Table 2); tube 7: Tx-RID 1 (Table 3); Tube 8: Positive control: 30 μL of 1fg/μL CL Brener *T*. *cruzi* stock (Tc VI) DNA.

*Tc* LAMP results obtained in this study were detected by naked-eye visualization, as exemplified in Fig 1.

#### Group I: Congenital CD (cCD)

*Tc* LAMP and qPCR detected *T*.*cruzi* DNA in nine VB and four DBS samples from all 13 cCD cases whereas VB samples from all 17 non-infected infants born to seropositive mothers were negatives (Table 1). Positive quantified blood samples had between 5.27 and 3063.47 par.eq/mL, using as a standard curve DNA obtained from seronegative blood spiked with CL Brener clone.

**Table 1:** Detection of *T*.*cruzi* DNA in infants born to seropositive mothers using *Tc* LAMP and qPCR.

Cases ID	Microscopy observation		Serological Findings			Gold standard Final Diagnosis	Treatment status	Molecular Analysis			
	Mtnity. Result (Age in days)	H. Ctr. Result (Age in days)	ELISA	HAI ¤	Final Result* (Age in days)			Type of Sample (Age in days)	LAMP Result	SatDNA qPCR Result	Parasitic Load** (p.e/mL)
cCD1	Nav	Neg (63)	0.759 Ɛ	64	Reactive (336)	cCD	Not treated	VB (336)	Pos	Pos	5.27
cCD2	Nav	Pos (87)	0.015 ß	NR	Non Reactive-treated (600)	cCD	Completed	VB (87)	Pos	Pos	3683.8
cCD3	Nav	Neg (36)	2.985 ß	256	Reactive (368)	cCD	Not treated	VB (36)	Pos	Pos	356.19
cCD4	Nav	Pos (10)	0.036 ¥	NR	Non Reactive-treated (275)	cCD	Completed	VB (10)	Pos	Pos	640.98
cCD5	Nav	Pos (49)	2.501 ß	256	Reactive (322)	cCD	Not treated #	VB (49)	Pos	Pos	37.47
cCD6	Nav	Pos (8)	2.624 §	256	Reactive (303)	cCD	Not treated ##	VB (303)	Pos	Pos	3063.47
cCD7	Neg (1)	Neg (41)	2.531 ß	128	Reactive (427)	cCD	Not treated	VB (41)	Pos	Pos	53.35
cCD8	NAv	Neg (45)	1.418 ¥	256	Reactive (331)	cCD	Not treated	VB (45)	Pos	Pos	81.96
cCD9	Pos (1)	Neg (44)	0.059 ¥	NR	Non Reactive-treated (324)	cCD	Completed	VB (44)	Pos	Pos	12.96
cCD10	Pos (2)	NA	Nav	NR	Non Reactive-treated (Nav)	cCD	Completed	DBS (2)	Pos	Pos	NA
cCD11	Pos (2)	NA	Nav	NR	Non Reactive-treated (Nav)	cCD	Completed	DBS (2)	Pos	Pos	NA
cCD12	Pos (2)	NA	Nav	NR	Non Reactive-treated (Nav)	cCD	Completed	DBS (2)	Pos	Pos	NA
cCD13	Pos (2)	NA	Nav	NR	Non Reactive-treated (Nav)	cCD	Completed	DBS (2)	Pos	Pos	NA
cCD14	Nav	Neg (10)	0.016 §	NR	Non Reactive (435)	Non Infected	NA	VB (435)	Neg	Neg	NA
cCD15	Nav	Neg (104)	0.036 ¥	NR	Non Reactive (306)	Non Infected	NA	VB (104)	Neg	Neg	NA
cCD16	Nav	Neg (112)	0.014 ß	NR	Non Reactive (312)	Non Infected	NA	VB (312)	Neg	Neg	NA
cCD17	Nav	Neg (64)	0.015 ß	NR	Non Reactive (350)	Non Infected	NA	VB (64)	Neg	Neg	NA
cCD18	Nav	Neg (40)	0.016 ß	NR	Non Reactive (319)	Non Infected	NA	VB (40)	Neg	Neg	NA
cCD19	Nav	Neg (30)	0.039 ¥	NR	Non Reactive (304)	Non Infected	NA	VB (304)	Neg	Neg	NA
cCD20	Neg (1)	Neg (31)	0.038 ¥	NR	Non Reactive (304)	Non Infected	NA	VB (31)	Neg	Neg	NA
cCD21	Neg (2)	Neg (33)	0.015 ß	NR	Non Reactive (306)	Non Infected	NA	VB (2)	Neg	Neg	NA
cCD22	Neg (1)	Neg (62)	0.022 ¥	NR	Non Reactive (321)	Non Infected	NA	VB (321)	Neg	Neg	NA
cCD23	Nav	Neg (122)	0.018 ß	NR	Non Reactive (326)	Non Infected	NA	VB (326)	Neg	Neg	NA
cCD24	Nav	Neg (60)	0.036 ¥	NR	Non Reactive (304)	Non Infected	NA	VB (304)	Neg	Neg	NA
cCD25	Nav	Neg (28)	0.014 ß	NR	Non Reactive (307)	Non Infected	NA	VB (28)	Neg	Neg	NA
cCD26	Neg (2)	NA	0.014 ß	NR	Non Reactive (305)	Non Infected	NA	VB (2)	Neg	Neg	NA
cCD27	Neg (2)	NA	0.012 ß	NR	Non Reactive (329)	Non Infected	NA	VB (2)	Neg	Neg	NA
cCD28	Neg (2)	NA	0.014 ß	NR	Non Reactive (346)	Non Infected	NA	VB (2)	Neg	Neg	NA
cCD29	Neg (2)	NA	0.017 ß	NR	Non Reactive (356)	Non Infected	NA	VB (2)	Neg	Neg	NA
cCD30	Neg (1)	NA	0.071¥	NR	Non Reactive (316)	Non Infected	NA	VB (1)	Neg	Neg	NA

^Ɛ^ cut-off: 0.152;

^§^ cut-off: 0.318;

^ß^ cut-off: 0.316;

^¥^ cut-off: 0.322;

¤ cut-off: 1

Mtnity: maternity service. H.Ctr: health center. M: male; F: female; MD: missing data; VB: venous blood containing 25% of DNAgard stabilizer; DBS: dried blood spot; Nav: sample not available; NA: not applicable; Pos: positive; Neg: negative; p.e/mL: parasite equivalents/milliliter.

* The criterion for serological reactivity was based on at least two positive serological tests.

** For PCR quantification, the standard curve consisted of DNA obtained from seronegative human blood spiked with known amounts of CL Brener cultured parasites (TcVI).

^#^ The patient did not complete treatment;

^##^ Comorbidities: bronchiolitis and nosocomial infection.

#### Group II: Orally-transmitted CD (oCD)

Guanidine hydrochloride-EDTA blood samples were available from six patients with oCD. Five of them were children and all six presented cardiac manifestations at the time they were diagnosed; echocardiograms showed pericardial effusion in all the children, but related symptoms, such as dyspnea or precordial pain, were not present. Their EKGs were abnormal, showing mainly alteration of the repolarization (Table 2).

**Table 2:** Detection of oral infection of *T*.*cruzi* by means of Tc LAMP and qPCR.

oCD case	Age (years)	Clinical manifestations	Echo / EKG	Tc LAMP	Sat qPCR DNA	
					Result	Load* (p.e/mL)
oCD1	10	Cardiac	Pericardial effusion/ Altered	Pos	Pos	10.44
oCD2	8	Cardiac	Pericardial effusion/ Altered	Pos	Pos	0.56
oCD3	9	Cardiac	Pericardial effusion/ Altered	Pos	Pos	122.72
oCD4	36	Cardiac/HIV seropositive	Pericardial effusion/ Altered	Pos	Pos	80.54
oCD5	7	Cardiac	Pericardial effusion/ Altered	Neg	Pos	0.003
oCD6	9	Cardiac	Pericardial effusion/ Altered	Pos	Pos	0.09

M: male; F: female; HIV: Human Immunodeficiency Virus; Echo: Echocardiogram; EKG: Electrocardiogram; Pos: positive; Neg: negative; p.e/mL: parasite equivalents/milliliter;

* For qPCR quantification, the standard curve was DNA from seronegative human blood spiked with known amounts of Silvio X 10 clone (TcI).

Five cases were concordantly positive by Tc LAMP and qPCR, while patient oCD5 was not detected by Tc LAMP. The sample showed only 0.003 par.eq/mL by qPCR, indicating very low parasitic load.

#### Group III: Recipients of organs from infected donors (Tx-RID)

Tc LAMP was tested in EDTA-blood samples from four seronegative recipients of organs explanted from seropositive donors. In these cases, qPCR was interpreted as indicator of infection. Except the sample from case Tx-RID 3, the other three were Tc LAMP positive, presenting parasitic loads ranging from 11.5 to 4060.5 par.eq/mL, using as a standard curve DNA obtained from seronegative blood spiked with CL Brener clone (Table 3). The discordant Tc LAMP negative and qPCR positive sample from case Tx-RID 3 had only 0.5 par.eq/mL, indicating very low parasitic load.

**Table 3:** Detection of acute infection in blood samples from seronegative recipients of organs from *T*.*cruzi* infected donors.

Case ID	Age (years)	Time after Tx (days)	Tx-organ	Receptor Serology	Organ Donor Serology	Tc LAMP	Sat qPCR	
							Result	Load* p.e/mL
Tx-RID 1	71	63	Liver	Non Reactive	Reactive	Positive	Positive	11.5
Tx-RID 2	20	MD	Kidney	Non Reactive	Reactive	Positive	Positive	2939.5
Tx-RID 3	63	MD	Liver- Kidney	Non Reactive	Reactive	Negative	Positive	0.5
Tx-RID 4	55	69	Liver	Non Reactive	Reactive	Positive	Positive	4060.5

M: male; F: female, MD: missing data; p.e/mL: parasite equivalents/milliliter

#### Group IV: Chagas disease and HIV coinfected patients (CD-HIV)

All six CD-HIV patients gave positive Tc LAMP and qPCR results in peripheral blood and/or CSF samples collected at the time CD reactivation was suspected (Table 4).

**Table 4:** Tc LAMP and qPCR detection of reactivation in CD-HIV patients.

**Case ID**	Age (years)	Clinical Features / CNS Images	CD4 (cells/mL)	Survival after one year	Parasitological findings	Sample type	Tc LAMP result	Satellite qPCR	
								Result	Load* (p.e/mL)
CD-HIV1	42	Seizures/encephalitis with two space-occupying lesions	7	No	Trypomastigotes (CSF)	EB	Pos	Pos	107
CD-HIV2	55	Sensory impairment/marked cerebral cortex atrophy; encephalitis with two space-occupying lesions	10	No	Trypomastigotes (CSF)	CSF	Pos	Pos	3511.5
						EB	Pos	Pos	143
CD-HIV3	39	Right hemiparesis, facio-brachio-crural/encephalitis with large space occupying lesion and brain midline shift	10	Yes	Strout positive	GEB	Pos	Pos	677
CD-HIV4	12	Non-Hodgkin Lymphoma/cardio-respiratory failure; without encephalitis	93	Yes	Strout positive	DBS	Pos	Pos	44
CD-HIV5	61	Space-occupying lesion/ vascular and non-vascular encephalic lesions in NMR	43	Yes	Strout negative	DBS	Pos	Pos	47.5
CD-HIV6	48	Space-occupying lesion/ multiple hypodense brain images on CAT scan	63	No	Strout positive	DBS	Pos	Pos	10.5

HIV: human immunodeficiency virus; M: male; F: female; CNS: central nervous system; CSF: cerebrospinal fluid; EB: EDTA-treated blood; GEB: guanidine EDTA-treated blood. DBS: Dried blood spot. p.e/mL: parasite equivalents/milliliter. NMR: Nuclear Magnetic Resonance. The criterion for risk of reactivation in HIV subjects is CD4 counts < 200 cells/mL

### Sensitivity, specificity and agreement

Fig 1 illustrates examples of Tc LAMP results obtained from blood specimens of study cases, visualized with the naked eye.

**Fig 2:**
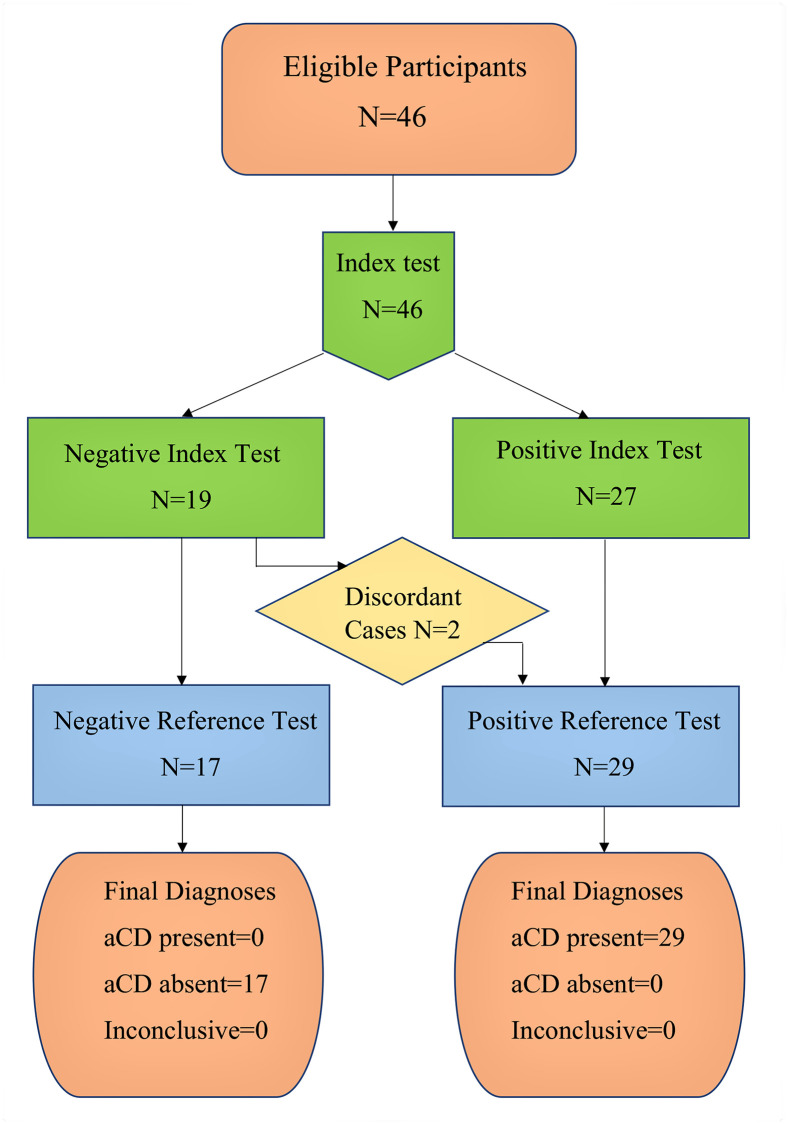
Flow of participants. aCD: acute Chagas disease; Index Test: Tc LAMP; Reference Test: qPCR.

The limited number of samples per group precluded estimating the accuracy of Tc LAMP for each group. Therefore, the sensitivity and specificity of Tc LAMP were estimated pooling the data obtained from the 46 blood samples: 29 samples from confirmed Chagas disease cases (13 cCD, 6 oCD, 4 Tx-RID, and 6 Chagas-HIV) and 17 samples from non-infected cases, all non-infected infants born to seropositive mothers. These results are summarized in Fig 2.

The sensitivity of Tc LAMP was 93% (95% CI: 77–99) and its specificity was 100% (95% CI: 80–100). The agreement between Tc LAMP and qPCR was almost perfect (κ = 0.92, 95% CI: 0.62–1.00) as only two samples that resulted in Tc LAMP negative were qPCR positive (oCD5, Table 2 and Tx-RID 3, Table 3.

## Discussion

Our approach to the evaluation of a LAMP assay for diagnosis of Chagas disease has been guided by the knowledge of the resource-limited settings where this neglected disease is still endemic as well as the insufficient investment of resources allocated to the control of CD transmission, including in non-endemic countries. Evidence clearly demonstrates that early diagnosis and treatment results in effective parasite eradication and a reduction in loss to follow-up of the patient. The *T*. *cruzi* Loopamp assay showed high values of sensitivity, specificity and agreement with qPCR in the following clinical settings:

i) Congenital infection: Even if the accuracy of Tc LAMP per clinical group is not presented, it should be noted that both the sensitivity and specificity of Tc LAMP in the cCD group were 100% (13 cases and 17 non-cases correctly diagnosed by Tc LAMP), with complete agreement with qPCR results (Table 1). The current parasitological/serological diagnostic algorithm remains unsatisfactory due to the poor sensitivity and operator dependence of parasitological methods, along with loss to follow-up, once the mother-newborn pair has left the maternity service. This loss to follow-up and diagnosis opportunity may reach 55–80% depending on the geographic area [20,31–35]. In our panel of samples, there were two cases (cCD3 and cCD7, Table 1) that had negative micromethod results with positive molecular findings in the same blood collection, indicating the higher sensitivity of molecular methods and the inadequate performance, due to operator dependency, of the parasitological method even in samples with high parasitic loads (cCD3: 356.19 par.eq/mL).

Several studies using different in-house PCR methods depicted better performance than the current diagnostic cCD algorithm [36–39]. However, evaluation of commercial methods suitable for clinical practice are urgently needed and there are only a few reports on the use of nucleic acid amplification kits [9,15,40,41]. Our data show the diagnostic potential of the Loopamp *Trypanosoma cruzi* prototype kit for early detection of congenital transmission.

ii) Orally-infected CD: This route of transmission, associated with the consumption of food contaminated with triatomine feces or didelphid secretions leading to high morbidity and mortality rates in endemic areas, has been remarkable in the last decade [16,42,43]. In most outbreaks, molecular tools have been fundamental for specific diagnoses [44–47]. The oral outbreak under study took place mainly in school populations from Chichiriviche de la Costa, a rural and touristic seashore community located on the central Venezuelan coast. It affected 89 persons, with 5 deaths. Frequency of symptomatic patients was high (89.9%), with long-standing fever in 87.5%; 82.3% had pericardial effusion detected by echocardiogram and 41% had EKG abnormalities [16]. The availability of a LAMP kit that could be used in health facilities close to rural areas where these oral outbreaks can take place would be of great benefit for rapid screening of people suspected of infection, thus allowing prompt treatment.

iii) In the immunocompromised condition, prompt treatment prevents life-threatening complications so early detection of infection is critical to improve patient management [4, 6, 48, 49]. *Tc* LAMP was able to detect parasite DNA in EDTA-blood, guanidine hydrochloride-EDTA blood, and dried blood spots collected in filter paper, as well as in CSF samples.

The accuracy of *Tc* LAMP to diagnose different acute forms of CD was excellent (93% sensitivity and 100% specificity). These estimates were obtained combining the results from samples of the different groups of patients (n = 46), as the number of samples analyzed per group was limited and only 17 of those were non-cases. The *Tc* LAMP clinical sensitivity and specificity estimates reported are important, as samples from acute CD cases have similar characteristics, i.e. high parasitemia and diagnostic approach (microscopy observation, PCR). Furthermore, the samples from these patients are rare and difficult to obtain and the results of this study prove the value of the *T*. *cruzi* Loopamp kit as a diagnostic tool for congenital, acute and CD reactivation cases. A Chagas disease surveillance study carried out in the endemic provinces of Northern Argentina showed 100% of specificity for *Tc* LAMP performed in blood samples from 59 non-infected seronegative adult participants (S1 Table). Another LAMP study on cCD samples showed high agreement between parasitological observation and *Tc* LAMP results [14].

In most of the samples, Tc LAMP and qPCR results were concordant. The two Tc LAMP-negative samples that were positive by qPCR presented parasitic loads close or below the analytical limit of detection of the methodology [10, 11]. *Tc* LAMP-negative DNA samples appeared adequate in integrity and did not contain inhibitors for qPCR as seen by amplification of the endogenous human RNase P gene (oCD group), β-actin PCR (DBS from cCD and CD-HIV cases) and IAC (rest of the Groups).

Our previous analytical study using this LAMP assay was carried out with purified DNA from reference *T*. *cruzi* strains belonging to the different DTUs and showed no significant differences in analytical sensitivity among them [15]. In the present work, we were able to detect infection in clinical samples that were shown to be infected by TcI (oCD Group) and TcV populations (cCD group) [29, 40].

Tc LAMP was carried out using DNA samples obtained from a variety of sample supports and stabilizing agents, such as frozen blood treated with EDTA or with GE buffer, blood treated with DNAgard stabilizer agent, DBS in Whatman filter paper used for the National Neonatal Screening Program, as well as from CSF specimens. It is expected that this technique will work also for testing skin, endomyocardial, and/or brain biopsy or necropsy tissue sections for rapid confirmation of presumptive CD reactivation [25]. The approximate cost of the Tc LAMP kit is at least one half of the cost of a qPCR kit. Its final cost will depend on the chosen DNA extraction method and on transportation and customs costs in the countries. As a result, the next steps towards optimization of standard operating procedures using Tc LAMP should include the development of more simple DNA extraction methods without loss of sensitivity, and field validation of the assay in the context of primary health centers and point-of-care laboratories [12,50].

### Conclusions

Tc LAMP is a potentially useful rapid laboratory tool for the diagnosis and treatment monitoring of *T*. *cruzi* acute infections and cases of reactivation. Its advantages are that no complex laboratory infrastructure is needed, allowing its application in healthcare facilities with limited equipment, and giving results rapidly through detection by naked eye.

## Supporting information

S1 Checklist STARD Checklist. (DOCX)

S1 Table *T*. *cruzi* Loopamp test on samples of non-infected individuals (NI). (DOCX)
