# Supplementary material for: Trypanosoma cruzi loop-mediated isothermal amplification (Trypanosoma cruzi Loopamp) kit for detection of congenital, acute and Chagas disease reactivation
Source: PLoS Negl Trop Dis. 2020 Aug 14;14(8):e0008402. doi: 10.1371/journal.pntd.0008402 (PMC7458301; doi:10.1371/journal.pntd.0008402)
Supplement: S1 Table — (DOCX) [file pntd.0008402.s002.docx]

**S1 Table:** ***T. cruzi* Loopamp™ test on samples of non-infected individuals (NI).**

EDTA- Blood samples were previously tested by two serological assays for Chagas Disease based on different methodologies.

| Case Code | Age (years) | Serology | LAMP Naked Eye | SatDNA qPCR |
| --- | --- | --- | --- | --- |
|  |  |  |  |  |
| NI 1 | 37 | NR | Neg | Neg |
| NI 2 | 20 | NR | Neg | Neg |
| NI 3 | 52 | NR | Neg | Neg |
| NI 4 | 55 | NR | Neg | Neg |
| NI 5 | 11 | NR | Neg | Neg |
| NI 6 | 48 | NR | Neg | Neg |
| NI 7 | 49 | NR | Neg | Neg |
| NI 8 | 68 | NR | Neg | Neg |
| NI 9 | 43 | NR | Neg | Neg |
| NI 10 | 29 | NR | Neg | Neg |
| NI 11 | 53 | NR | Neg | Neg |
| NI 12 | 19 | NR | Neg | Neg |
| NI 13 | 14 | NR | Neg | Neg |
| NI 14 | 23 | NR | Neg | Neg |
| NI 15 | 59 | NR | Neg | Neg |
| NI 16 | 80 | NR | Neg | Neg |
| NI 17 | 17 | NR | Neg | Neg |
| NI 18 | 42 | NR | Neg | Neg |
| NI 19 | 51 | NR | Neg | Neg |
| NI 20 | 30 | NR | Neg | Neg |
| NI 21 | 60 | NR | Neg | Neg |
| NI 22 | 16 | NR | Neg | Neg |
| NI 23 | 50 | NR | Neg | Neg |
| NI 24 | 55 | NR | Neg | Neg |
| NI 25 | 42 | NR | Neg | Neg |
| NI 26 | 14 | NR | Neg | Neg |
| NI 27 | 6 | NR | Neg | Neg |
| NI 28 | 7 | NR | Neg | Neg |
| NI 29 | 20 | NR | Neg | Neg |
| NI 30 | 18 | NR | Neg | Neg |
| NI 31 | 17 | NR | Neg | Neg |
| NI 32 | 17 | NR | Neg | Neg |
| NI 33 | 60 | NR | Neg | Neg |
| NI 34 | 25 | NR | Neg | Neg |
| NI 35 | 16 | NR | Neg | Neg |
| NI 36 | 26 | NR | Neg | Neg |
| NI 37 | 25 | NR | Neg | Neg |
| NI 38 | 35 | NR | Neg | Neg |
| NI 39 | 70 | NR | Neg | Neg |
| NI 40 | 33 | NR | Neg | Neg |
| NI 41 | 76 | NR | Neg | Neg |
| NI 42 | 60 | NR | Neg | Neg |
| NI 43 | 45 | NR | Neg | Neg |
| NI 44 | 42 | NR | Neg | Neg |
| NI 45 | 53 | NR | Neg | Neg |
| NI 46 | 19 | NR | Neg | Neg |
| NI 47 | 55 | NR | Neg | Neg |
| NI 48 | 35 | NR | Neg | Neg |
| NI 49 | 78 | NR | Neg | Neg |
| NI 50 | 75 | NR | Neg | Neg |
| NI 51 | 60 | NR | Neg | Neg |
| NI 52 | 52 | NR | Neg | Neg |
| NI 53 | 35 | NR | Neg | Neg |
| NI 54 | 33 | NR | Neg | Neg |
| NI 55 | 54 | NR | Neg | Neg |
| NI 56 | 55 | NR | Neg | Neg |
| NI 57 | 72 | NR | Neg | Neg |
| NI 58 | 52 | NR | Neg | Neg |
| NI 59 | 36 | NR | Neg | Neg |

**NI: non infected; NR: non reactive; Neg: negative.**
